# Anatomical and Neurochemical Correlates of Parental Verbal Abuse: A Combined MRS—Diffusion MRI Study

**DOI:** 10.3389/fnhum.2019.00012

**Published:** 2019-01-29

**Authors:** Dohyun Kim, Jae Hyun Yoo, Young Woo Park, Minchul Kim, Dong Woo Shin, Bumseok Jeong

**Affiliations:** ^1^Graduate School of Medical Science and Engineering, Korea Advanced Institute for Science and Technology (KAIST), Daejeon, South Korea; ^2^School of Electrical Engineering, Korea Advanced Institute of Science and Technology (KAIST), Daejeon, South Korea

**Keywords:** verbal abuse, frontolimbic circuit, magnetic resonance spectroscopy, diffusion tensor imaging, probabilistic tractography, pregenual anterior cingulate cortex, maltreatment

## Abstract

Despite the critical impact of parental dialog on children who remain physically and psychologically dependent, most studies have focused on brain alterations in people exposed to moderate-to-high levels of emotional maltreatment with/without psychopathology. We measured metabolites in the pregenual anterior cingulate cortex (pgACC) acquired with single-voxel proton magnetic resonance spectroscopy and anatomical connectivity assessed with probabilistic tractography in 46 healthy young adults who experienced no-to-low level parental verbal abuse (paVA) during their childhood and adolescence. The partial least square regression (PLSR) model showed that individual variance of perceived paVA was associated with chemical properties and structural connectivity of pregenual anterior cingulate cortex (pgACC; prediction *R*^2^ = 0.23). The jackknife test was used to identify features that significantly contributed to the partial least square regression (PLSR) model; a negative association of paVA was found with myo-inositol concentration, anatomical connectivities with the right caudate and with the right transverse temporal gyrus. Of note, positive associations were also found with the left pars triangularis, left cuneus, right inferior temporal cortex, right entorhinal cortex and right amygdala. Our results showing both a negative association of frontal glial function and positive associations of anatomical connectivities in several networks associated with threat detection or visual information processing suggest both anatomical and neurochemical adaptive changes in medial frontolimbic networks to low-level paVA experiences.

## Introduction

As children and adolescents remain physically and psychologically dependent, maltreatment by caregivers can have considerable consequences in both psychosocial development and brain development. A survey of 4,141 participants reported that exhibiting a greater number of lifetime anxiety disorders is associated with a higher likelihood of childhood physical and sexual abuse history (Cougle et al., 2010). In a 32-year prospective longitudinal study, exposure to adverse psychosocial experiences such as maltreatment elevated the risk of depression in adulthood (Danese et al., 2009). In a prior study (Danese et al., 2009), incidence rate ratios indexing the association between depression risk in adulthood and childhood maltreatment were 1.81 in the definite group (having experienced two and more among five maltreatment indicators) and 1.37 in the probable group (having experienced one indicator of maltreatment). Furthermore, depression (Hovens et al., 2012; Nanni et al., 2012) and bipolar disorder (Post et al., 2015) emerge earlier in maltreated individuals, and these individuals have a more sustained or difficult treatment course. As childhood maltreatment precedes clinical states and occurs in developmental periods, one possible hypothesis is that such maltreatment experiences can modulate the structure of a person’s model regarding the external world and one’s self. This modulation could be reflected in the structure and function of the brain, as reported in previous studies with healthy subjects (Choi et al., 2009; Lee et al., 2015). Thus, the difficult progression of mood disorders in maltreated individuals may be more likely associated with aberrancy of a person’s inner model for perception of the world rather than simply with the comorbidity of two conditions (e.g., The world around me is not safe and requires high surveillance. Life is hard on me.).

Previous neuroimaging studies in clinical patients having experienced physical or emotional maltreatment have reported anatomical, functional, and neurochemical changes in the brain. A multimodal study of bipolar disorder showed the association of severity of childhood trauma with altered prefrontolimbic functional connectivity and uncinate fasciculus fractional anisotropy (Souza-Queiroz et al., 2016). A magnetic resonance spectroscopy (MRS) study in maltreated children and adolescents with posttraumatic stress disorder (PTSD) reported a decreased N-acetyl aspartate (NAA) to creatine (Cr) ratio of the anterior cingulate cortex, indicative of neuronal loss or neuronal dysfunction (De Bellis et al., 2000). In a functional magnetic resonance imaging (fMRI) study, emotionally neglected adolescents showed blunted ventral striatum development, which predicted the emergence of depressive symptoms (Hanson et al., 2015). Regardless of comorbid psychopathology, subjects with moderate-to-high maltreatment have decreased intermodular connections in terms of network architecture (Ohashi et al., 2017). These anatomical and functional changes were also reported in healthy subjects with a history of childhood maltreatment. Young adults with parental verbal abuse (paVA) history but without significant psychopathology showed decreased fractional anisotropy in the arcuate fasciculus, the fornix, and the cingulum bundle (Choi et al., 2009) and larger gray matter (GM) volume of the left primary auditory cortex (Tomoda et al., 2011). The degree of peer verbal abuse was negatively correlated with the white matter (WM) integrity of the corpus callosum and the corona radiata (Teicher et al., 2010). Functional connectivity between the right amygdala and the rostral anterior cingulate during processing of negative emotion was associated with previous verbal abuse experience (Lee et al., 2015). This alteration of the frontolimbic circuit during emotional processing was also reported in a longitudinal sample of adults (aged from 23 to 37 years) having experienced one or more forms of maltreatment from among emotional maltreatment, physical neglect, physical abuse and sexual abuse (Jedd et al., 2015).

Neuroimaging results from previous studies in subjects with or without comorbid psychiatric disorders indicate that childhood maltreatment experiences are associated with anatomical, functional, and neurochemical alterations in the brain, including ventromedial and orbitofrontal-limbic networks, and have deleterious effects on psychopathology (Teicher and Samson, 2016). However, unless comorbid psychiatric disorders are controlled, it is difficult to disentangle which of the changes in the brain are due to maltreatment, the associated psychiatric conditions or a combination or interaction of both (Hart and Rubia, 2012).

Not all persons with a history of maltreatment experience problematic outcomes. Although there is controversy over the interpretation of maltreatment-related brain alterations, experience-dependent plastic adaptation to a threatening environment can be a plausible interpretation as well as diathesis-stress mechanism (Teicher et al., 2016). Teicher et al. (2016) suggested that alterations of frontolimbic regions in healthy subjects with maltreatment experiences may be associated with altered models of perception of the external world, such as threat detection, sensory filtering, and reward processing. Regarding previous results showing anatomical, functional, and neurochemical alterations in frontolimbic regions, evaluating the whole-brain connectivity with core brain regions and chemical properties might be beneficial to elucidate the impact of maltreatment experiences on the brain. In particular, the pregenual anterior cingulate cortex (pgACC) is highly connected with the limbic system and is thought to be one of the key regulatory regions of the frontolimbic circuit, which is implicated in emotional processing and reward processing (Etkin et al., 2011; Marusak et al., 2016). The connectivity-based segmentation of the cingulate cortex allows the identification of structural connectivity between the cingulate and the rest of the brain (Beckmann et al., 2009). Measuring the chemical concentration in a predefined volume of interest (VOI), such as the pgACC, using single-voxel proton magnetic resonance spectroscopy (^1^H-MRS), can provide additional information that differs from that derived from diffusion tensor image (DTI). Thus, an integrated multimodal approach with ^1^H-MRS metabolites and anatomical connectivity of the pgACC might be useful to investigate brain changes in healthy young adults with a history of emotional maltreatment, such as verbal abuse.

In the current study, we investigated whether variance in the perceived intensity of paVA during childhood and adolescence in healthy young adults with a low level of paVA is associated with both anatomical connectivity of the pgACC to the whole brain and the chemical properties of the pgACC, which is a crucial region of the frontolimbic circuit (Etkin et al., 2011). We assumed that significant predictors would show both a positive association and a negative association if neural alterations to a low level of paVA resulted from maltreatment-related adaptation. In line with the adaptation hypothesis, clinical symptoms such as alexithymia or depression scores may not be able to be predicted with neural features.

Considering the maltreatment-related adaptation hypothesis, it is more likely that depending on the function of the tract, WM integrity may increase for some tracts, while WM integrity may decrease for other tracts. In functional studies, for example, a study on reward processing reported decreased striatum activity in maltreated subjects (Dillon et al., 2009), while another study reported a maltreatment-related amygdala hyperresponse during negative emotional processing (Dannlowski et al., 2012). However, most previous studies on WM alterations in maltreated subjects have reported a reduction in the number of fiber streams (Ohashi et al., 2017) or decreased WM integrity assessed by decreased fractional anisotropy or increased radial diffusivity (Eluvathingal et al., 2006; Choi et al., 2009; Rodrigo et al., 2016). Greening and Mitchell (2015) reported a positive association between structural connectivity and trait anxiety using seed-based probabilistic tractography. Compared with the whole-brain approach, the seed-based connectivity approach can provide specific information of tracts (Greening and Mitchell, 2015). To achieve our goals, we exploited partial least square regression (PLSR) with data acquired from probabilistic tractography and ^1^H-MRS of the pgACC. Because recent studies described a reliable prediction model for various dimensions of psychiatric symptoms or cognitive performance using anatomical or functional connectivity (Greening and Mitchell, 2015; Meskaldji et al., 2016; Rosenberg et al., 2016; Yoo et al., 2018), we focused on the effect of paVA rather than on effects of confounding factors such as current psychopathology on the medial frontolimbic circuit.

## Materials and Methods

### Participants

We recruited 51 young adults from Korea Advanced Institute of Science and Technology (KAIST). The subjects were interviewed by skilled psychiatrists (DK and JY) using the Korean version of the Diagnostic Interview for Genetic Studies (DIGS-K version 2.0; Joo et al., 2004). Two subjects were excluded because of incomplete self-report questionnaires and structured interviews. The remaining subjects had no history of psychiatric or neurological illness or of other types of abuse, such as physical abuse or sexual abuse. Subjects also underwent MRI scans consisting of T1-weighted (T1w) imaging, ^1^H-MRS, DTI, and resting and task-related fMRI (fMRI data are not reported here). After the quality control process, an additional three subjects were excluded because of excessive motion on DTI (*N* = 2) and poor spectrum fitting of ^1^H-MRS (*N* = 1; see section “Quality Control” in the “Materials and Method” section). Finally, 46 subjects (mean age = 24.1 ± 4.2 years, male:female = 29:17, total intelligence quotient = 120.5 ± 9.6) were included in the further analysis. All procedures performed in this study involving human participants were in accordance with the ethical standards of the Institutional Review Board of KAIST and with the 1964 Helsinki declaration and its later amendments or comparable ethical standards. All participants signed informed consent. The Korean version of the verbal abuse questionnaire (VAQ; Teicher et al., 2006; Jeong et al., 2015) was used to measure the perceived severity of paVA experiences during the subjects’ childhood and adolescence. The VAQ is composed of 15 items covering scolding, yelling, swearing, blaming, insulting, threatening, demeaning, ridiculing, criticizing, and belittling. Perceived severity was reported using an 8-point Likert scale from 0 (not at all) to 7 (everyday). The Korean version of the VAQ was successfully used in our previous studies for adolescents and young adults (Jeong et al., 2015). A VAQ score above 40 delineates a level of verbal abuse that is unusually high in both North American and Korean populations. The group averaged VAQ score was 7.0 (SD = 6.7, range = 0–29). Accompanying psychiatric disorders could make difficult to disentangle which of the changes of the brain are due to maltreatment or associated psychiatric conditions. The recruitment of subjects with high VAQ scores but without an accompanying psychiatric disorder could possibility introduce a type of selection bias considering the aspect of resilience. Thus, here, we enrolled subjects with VAQ scores below 40. There was no gender difference in the VAQ score (Mean_male_ = 6.6, Mean_female_ = 7.7, *t* = −0.56, *p*-value = 0.57). Because self-report questionnaires including the VAQ rely on recall and subjectivity, such questionnaires may reflect a person’s inner model for perception of their maltreatment-related experience rather than accurate intensity of maltreatment. Thus, we assumed that the VAQ score represents the perceived intensity of paVA experiences in their childhood and adolescence. In addition to the VAQ, subjects were also asked to fill out self-report questionnaires such as the Center for Epidemiological Studies–Depression (CES-D, mean ± SD = 9.33 ± 6.8) questionnaire and the Toronto alexithymia scale (TAS, mean ± SD = 43.7 ± 10.4) questionnaire.

### MRI and MRS: Data Acquisition

**Figure 1:**
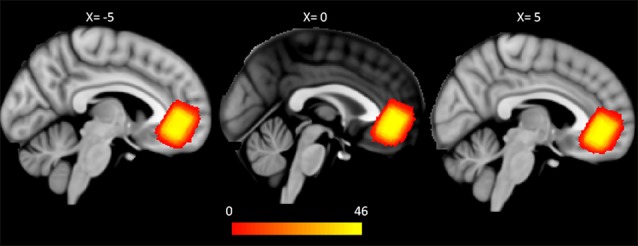
Group-wise pgACC VOI for magnetic resonance spectroscopy (MRS). Each binary mask for the pgACC VOI was transformed from native T1 space to the MNI 1 mm space. The VOIs partly overlap the mOFC and the rACC on the DKT atlas in all of the subjects. The color bar represents the number of overlapped VOIs of the subjects (pgACC, pregenual anterior cingulate cortex; VOI, volume of interest; mOFC, medial orbitofrontal cortex; rACC, rostral anterior cingulate cortex; DKT, Desikan-Killiany-Tourville).

Data acquisition was performed using a Siemens 3T Verio scanner (Erlangen, Germany) at KAIST. High resolution T1w structural images (MPRAGE, TR/TE/TI: 2,400 ms/2.02 ms/1,000 ms, flip angle: 8°, FOV: 224 × 224 mm, voxel size: 0.7 × 0.7 × 0.7 mm^3^) were acquired from the participants. After structural image acquisition, shimming was performed using FASTESTMAP, and water suppression pulses were calibrated for ^1^H-MRS. Single-voxel ^1^H-MRS using semilocalization by adiabatic selective refocusing (sLASER, TR/TE = 5,000 ms/28 ms, 64 scans, 2,048 complex points were acquired from VOI (size = 20 × 15 × 20 mm^3^) within pgACC (Figure 1), which was manually positioned by a psychiatrist or a neurologist (DK, JY, and DS). The sLASER sequence has advantages in terms of maintaining a relatively uniform B1 field and a desired flip angle within voxels (Zhu and Barker, 2011). In the sagittal view, the VOI was placed at the front of the genu of the corpus callosum. In coronal and axial views, the VOI was positioned at the midline of the pgACC to cover the maximum volume of GM in the region (Figure 1). Unsuppressed water signals were also collected to preprocess the data, such as eddy current and phase correction.

Participants additionally underwent DTI scanning. The diffusion images were acquired with a multiband protocol (TR/TE: 5,520 ms/94.2 ms, voxel size: 2 × 2 × 2 mm^3^, multiband factor = 4). The acquired multi *b*-value diffusion-weighted images consisted of two B0 images (b factor = 0 s/m^2^) with opposite phase encoding directions (from right to left and from left to right) and 30 diffusion-weighted images for each *b*-value (1,000, 1,500, 2,000 s/m^2^).

### MRI and MRS Data Preprocessing

#### Quality Control

The diffusion data were corrected for motion artifacts and eddy current-induced distortion by using the *topup* (Andersson et al., 2003) and *edd*y (Andersson and Sotiropoulos, 2016) functions of the FMRIB Software Library (FSL^1^). To estimate the susceptibility off-resonance field, two B0 images with right-to-left and left-to-right phase encoding directions were used.

The preprocessing pipeline for ^1^H-MRS data consisted of eddy current correction, frequency correction using a cross-correlation algorithm and phase correction using the least square algorithm, part of MRspa software (version 1.5e^2^). Taking into account the CSF fraction within the VOI, the metabolite concentrations were estimated using LCModel software version 6.3-1J (Provencher, 1993) and a basis set (Deelchand et al., 2015). The estimates with Cramèr-Rao lower bounds (CRLB) of less than 20% were considered reliable (Ip et al., 2017). Five of the metabolites—NAA + NAA-gluatamate (total NAA), myo-inositol, glutamate + glutamine (Glx), total choline (Glycerylphosphorylcholine + Phosphorylcholine), and total Cr (Cr + phosphocreatine)—met the CRLB criterion (Supplementary Table S1 in Supplementary Material).

Visual inspection and preprocessing step described above, we conducted a further investigation for quality control. We investigated the LCModel fitting signal-to-noise ratio (SNR) and motion parameters for the MRS and diffusion data, respectively. In the diffusion data, motion parameters were defined as root mean squares (RMS) of displacements across intracerebral voxels (Andersson and Sotiropoulos, 2016). Among the RMS of motion of 90 diffusion-weighted images (30 directions for each *b*-value), we took the maximum value as the motion parameter of each subject. One subject was excluded due to an LCModel signal-to-noise ratio (SNR) <10, and two subjects were excluded due to a maximum RMS of motion >3 (Supplementary Figure S1 in Supplementary Material).

#### Anatomical Consistency Among Individuals’ VOIs

In this study, VOIs of ^1^H-MRS were used not only as positions for metabolite quantification but also as seeds for probabilistic tractography. Thus, the anatomical consistency of VOIs across subjects must be assessed. Transformation matrices for each subject were acquired for transformation of B0 to T1w images to the standard 1 mm^3^ Montreal Neurological Institute (MNI) space using Advanced Normalization Tools (ANTs^3^). Then, each subject’s VOI was transformed from the subject’s T1w space to the standard space using the transformation matrix. Finally, the generalized dice similarity coefficient as a measure of overlap was computed from the intersection and union of VOIs (Crum et al., 2006; Park et al., 2018). The generalized dice coefficient of VOIs of 46 subjects was 0.71 (Figure 1). Because a dice similarity coefficient of 0.7 or greater is regarded as excellent agreement for similarities between pairs (Zou et al., 2004; Crum et al., 2006), our data can be considered reliable for anatomical consistency.

#### Network Construction With Probabilistic Tractography From pgACC VOI to Whole Brain

Individual cortical Desikan-Killiany-Tourville (DKT) and subcortical parcellation images, which consist of 76 regions (Supplementary Table S2 in Supplementary material), were obtained from T1w images using the pipeline of the Freesurfer software^4^. Then, the parcellated image in each subject was transformed to the DTI space using the transformation matrix acquired from the ANTs algorithm. Using the GM-WM boundary shell (Bonilha et al., 2015), target masks were defined as the boundary shell within the cortical DKT atlas and the subcortical atlas. Seed masks were defined as voxels in the VOI that overlapped with the boundary shell. Among the 76 DKT target regions, the medial orbitofrontal cortex (mOFC) and the rostral cingulate cortex of the bilateral hemisphere were excluded because the seed regions (i.e., VOI for MRS) occupied the two regions bilaterally in 46 subjects. After Bayesian estimation of diffusion parameters at each voxel of the diffusion image exploiting FSL bedpostX, probabilistic tractography (Behrens et al., 2007) was performed in the diffusion space. To determine the connectivity map from the pgACC VOI with the rest of the brain, we adapted the method introduced by Greening and Mitchell (2015). Connectivity frequency maps to the target regions were generated while avoiding the ventricle mask of each subject. To generate the frequency map, 25,000 samples were initiated within the seed voxels (step length = 0.5, step size = 1,000, curvature threshold = 0.2). Finally, we generated 72 likelihood maps of seed-target connectivity for each subject and then took the average value within the connectivity likelihood map of each target region of interest (ROI) in the subject diffusion space for the connectivity feature of the prediction model. The processing step of diffusion data is summarized in Supplementary Figure S2 in Supplementary Material.

### Statistical Analysis

The analysis was performed using 72 connectivity and five chemical features. For problems with high dimension (p) and small sample size (n) for which *p* > *n*, PLSR is useful for predicting behavior from neuroimaging data (Krishnan et al., 2011). We used the PLSR algorithm (for more details, see supplementary methods in Supplementary Material) implemented in the *pls* package (Mevik and Wehrens, 2007) of R 3.4.3 software^5^. PLSR benefits from dimension reduction by using few components (Meskaldji et al., 2016). Compared with principal component analysis, PLSR finds components from a predictor, which can predict dependent variables (Abdi, 2010). All features were scaled across subjects (features were converted to value between 0 and 1) within the training set using the “scale” option in the *plsr* function in the *pls* package. To find the optimal number of components, bootstrap resampling was performed 100,000 times. For each iteration, PLSR was performed using a bootstrapped sample, and then the root mean squared error of prediction (RMSEP) of each number of components from 0 to 20 was obtained. RMSEP corresponding to each number of components was defined as the median value of RMSEP values acquired from 100,000-time resampling.

**Figure 2:**
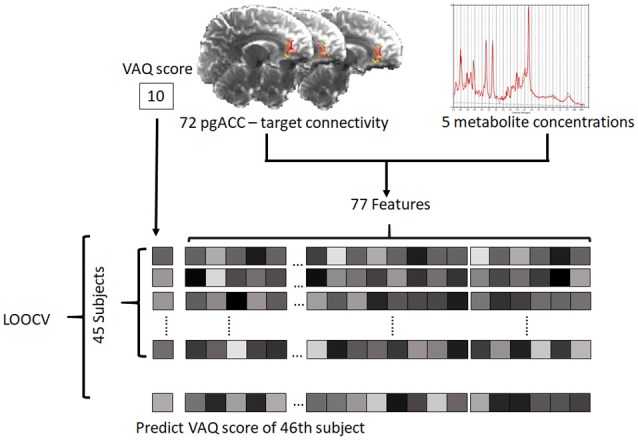
Schematic of the prediction model. For each subject, 72 connectivity features and five metabolite features were defined as predictors. The predictive model was trained on data from 45 subjects and tested with the remaining subject. The test was performed iteratively for all subjects (pgACC, pregenual anterior cingulate cortex; LOOCV, leave-one-out cross validation; VAQ, verbal abuse questionnaire).

The PLSR model with the optimal number of components was built on data from 45 subjects and tested with the remaining one subject [i.e., leave-one-out cross validation (LOOCV)], and this process was repeated for all 46 subjects (Figure 2). To evaluate the significance of the model, a correlation analysis between the reported VAQ value and the predicted VAQ value was performed. We repeated the evaluation process for 100,000 permutations, shuffling the VAQ scores across the subjects to estimate the significance of the PLSR model. For each permutation, Pearson’s correlation coefficient (*r*) between the predicted and shuffled VAQ score was obtained to generate a nonparametric distribution. The significance was evaluated by: (1) ranking the coefficient value of the PLSR model using the original data among the generated distribution; and (2) estimating the cross-validated prediction *R*^2^, which is given by

$$R^{2}=1-\;\frac{\text{Residual estimated sum of square for prediction (PRESS)}}{\text{Total sum of square (SST)}}$$

We compared the predictive ability of two PLSR models each incorporating a single modality (MRS alone and tractography alone) with the predictive ability of the multimodality PLSR model. Then, we use the jackknife test to approximate the *t*-test of the regression coefficient for the identification of significant features. The significance of features was determined at a *p*-value < 0.05. We performed additional PLSR using CES-D or TAS as a dependent variable to explore whether brain alteration is associated with psychopathology, such as depression or alexithymia. In the PLSR, CES-D score and TAS score were used as dependent variables, and both the connectivity features of 72 variables and the chemical features of five variables were used as independent variables, similar to the PLSR model for the VAQ score described above.

Furthermore, the prediction results were validated using different cross-validation schemes (i.e., five-fold, and 10-fold cross validation). Because the number of total subjects (*N* = 46) is not a multiple of 5 or 10, the subjects were divided into six groups of five subjects and four groups of four subjects for 10-fold cross validation and divided into one group of 10 subjects and four groups of nine subjects for five-fold cross validation. There are a number of ways to divide subjects into training sets and test sets; therefore, we repeated the process 10,000 times. The accuracy of the prediction was assessed using the mean of the cross-validated prediction *R*^2^ for each process.

## Results

### Prediction Performance of the Model

**Figure 3:**
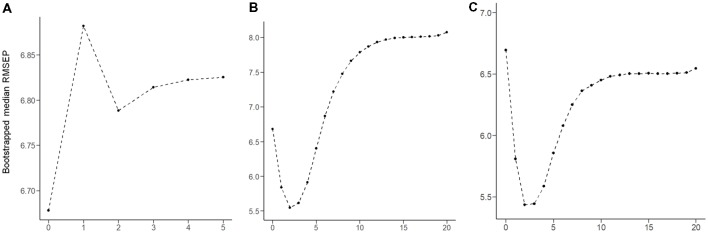
Root mean squared error of prediction (RMSEP) plot produced for 100,000 bootstrap resampling iterations. For each iteration, RMESP values of the partial least square regression (PLSR) model were obtained. The median RMSEP corresponding to each component is depicted.** (A)** PLSR model trained on the chemical properties of five features. **(B)** PLSR model trained on the connectivity of 72 features. **(C)** PLSR model trained on 77 features of both connectivity and chemical properties.

**Figure 4:**
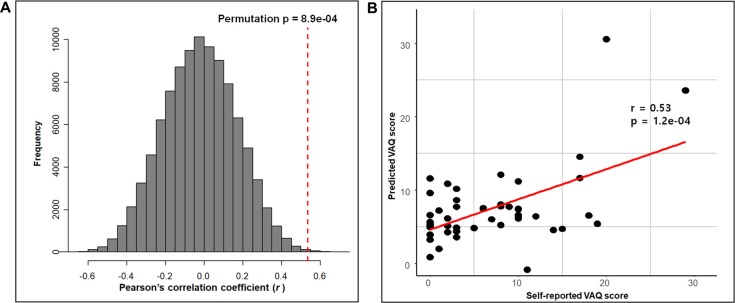
Prediction performance of the PLSR model with both connectivity and chemical properties of the pgACC. **(A)** The performance of the PLSR model tested by comparing the models trained on the shuffled data with 100,000 iterations. The red-dashed line represents the correlation coefficient between the self-reported VAQ score and the predicted VAQ score. For each iteration, the correlation coefficient between the shuffled VAQ score and the predicted VAQ score trained on the shuffled data was calculated. **(B)** Scatter plot of the self-reported VAQ score and the predicted VAQ score using the model. Predicted values were obtained using leave-one-out testing. We perform a correlation analysis between the predicted value and the self-reported value. The correlation coefficient was 0.53 (*p* = 1.2e-04).

The RMSEP plot represents the optimal number of components of the three PLSR models that include chemical properties, anatomical connectivity, and both anatomical connectivity and chemical properties, respectively (Figure 3). According to the plots, the optimal number of model components, corresponding to the lowest RMSEP value, was 2 in all three models. The model with two components of anatomical connectivity and the MRS chemical property of pgACC VOIs significantly outperformed the models trained on shuffled data (permutation *p* = 8.9 × 10^−4^, Figure 4). Pearson’s correlation between the predicted VAQ and the self-reported VAQ was significant (*r* = 0.53, *p* = 1.2 × 10^−4^, Figure 4), and the estimated prediction *R*^2^ was 0.23 (for validation test of the relationship, see Supplementary Material). The *R*^2^ of the PLSR model trained on anatomical connectivity alone was 0.20, while the PLSR model trained on chemical properties did not have predictive ability (*R*^2^ = −0.18*)*. TAS and CES-D scores could not be predicted using the PLSR models trained on pgACC connectivity or chemical properties (*R*^2^ = −0.35 and −0.56, respectively, Supplementary Figure S3 in Supplementary Material).

### Significant Features

**Figure 5:**
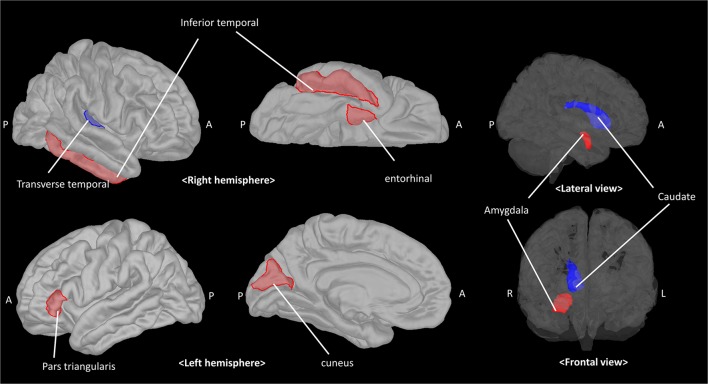
The pgACC connectivity features contributing to predicting the parental verbal abuse (paVA) score in the PLSR model. Red and blue patches represent target brain regions with significantly positive and negative coefficients, respectively. The estimated coefficient for each target is described in Table 1 (pgACC, pregenual anterior cingulate cortex; PLSR, partial least square regression; A, anterior; P, posterior; R, right; L, left).

Given that the model could predict paVA experience from the connectivity and chemical properties of the pgACC VOI, we sought to determine which features make a reliable contribution to the PLSR model. To identify these features, we used the jackknife test (for more details, see Supplementary Material) to estimate t-scores and *p*-values corresponding to the coefficients of features (Figure 5, Table 1, Supplementary Table S3 and Supplementary Figure S4 in Supplementary Material). The jackknife test showed that a high paVA score was negatively associated with pgACC connectivity with the right caudate, pgACC connectivity with the right transverse temporal cortex and myo-inositol concentration. Additionally, we found that pgACC connectivity with the left pars triangularis, with the left cuneus, with the right inferior temporal cortex, with the right entorhinal cortex and with the right amygdala were positively associated with VAQ score.

**Table 1:** Features significantly contributing to the model for predicting perceived parental verbal abuse.

Features	Coefficient	Standard error	*t*-value	*p*-value
Right caudate	−0.780	0.366	−2.166	0.045
Right transverse	−0.719	0.345	−2.118	0.042
temporal cortex
Myo-inositol	−0.768	0.335	−2.13	0.026
Right entorhinal	0.620	0.236	2.591	0.014
Left pars triangularis	0.703	0.212	3.258	0.003
Right inferior	0.781	0.323	2.377	0.024
temporal cortex
Left cuneus	0.764	0.37	2.086	0.044
Right amygdala	0.808	0.322	2.478	0.018

### Validation of the Prediction Model Using Different Cross-Validations

The mean cross-validated prediction *R*^2^ was 0.05 [range: −3.74 to 0.41] and 0.16 [range: −1.92 to 0.39] for five-fold cross-validation and 10-fold cross-validation, respectively. Five-fold cross-validation had 7,818 predictable cases (*R*^2^ > 0) and 10-fold cross validation had 9,636 predictable cases (*R*^2^ > 0) among the 10,000 iterations. A histogram of prediction *R*^2^ values in each validation procedure (Supplementary Figure S5 in Supplementary Material) showed that prediction accuracy was also maintained when different cross-validation schemes were used.

## Discussion

Using multimodal neuroimaging data consisting of the structural connectivity and chemical properties of the pgACC, we found significant association between perceived paVA and frontolimbic properties (cross-validated prediction *R*^2^ = 0.23). On the other hand, psychopathology scores, such as depressive symptoms (CES-D) or alexithymia (TAS), were not associated with the frontolimbic alterations. In other words, these structural and chemical alterations of the brain may reflect perceived paVA rather than psychopathology. These findings suggest that the significant neurobiological features found in our study may be attributed to individual experiences, such as perceived intensity to paVA experience.

The significantly contributing predictors in our study were consistent with the findings of previous neuroimaging studies of mentally healthy subjects (Choi et al., 2009; Dannlowski et al., 2012; Lee et al., 2015). Specifically, our results showing negative associations with perceived paVA suggest that more verbal abuse experience is associated with altered glial function indicated by lower myo-inositol concentration (Chang et al., 2013) and reduced pgACC connectivity with regions associated with auditory processing, the transverse temporal gyrus, and regions associated with reward processing, such as the caudate. Although most prior studies on maltreatment have reported a negative relationship between WM connectivity and the severity of maltreatment (Eluvathingal et al., 2006; Choi et al., 2009, 2012; Rodrigo et al., 2016), few studies reported positive association between maltreatment and WM tract (Hanson et al., 2013; Ugwu et al., 2015). We also found predictors showing a positive association with perceived paVA. Interestingly these predictors indicate connectivity of the pgACC with regions involved in threat detection, such as the amygdala, and with alertness, such as the cingulate-inferior frontal gyrus pathway (Sadaghiani and D’Esposito, 2015; Coste and Kleinschmidt, 2016). These findings revealing alterations of the frontolimbic region even in subjects with a mild intensity of perceived paVA below 40 suggest that the effect of maltreatment on the frontolimbic circuit is an adaptation to the external environment.

Many recent studies have attempted to predict cognitive performance or psychopathology using connectivity features (Greening and Mitchell, 2015; Rosenberg et al., 2016; Yoo et al., 2018). The significant prediction features not only contribute to accurate prediction but also demonstrate neurobiological background. Greening and Mitchell (2015) reported a prediction model for trait anxiety using the structural connectivity value from the amygdala to the rest of the brain based on the neurobiological background of anxiety. Because structural connectivity can be indicative of functional connectivity and can be affected by experience (May, 2011; Hermundstad et al., 2013), the perceived intensity of paVA can be reliably predicted using the pgACC connectivity value alone. However, the PLSR model trained on both connectivity and metabolite concentration offered a slight numerical advantage over the PLSR model trained with a single modality. Chemical properties can also be affected by psychiatric disorders and experiences and are associated with brain structure (Coupland et al., 2005; Zheng et al., 2010; Forde et al., 2018). Chemical properties also provide additional information, such as information on glial function and cytoarchitecture (Chang et al., 2013; Forde et al., 2018), compared with DTI tractography. Therefore, chemical properties may provide more neurobiological information and contribute to the accuracy of the prediction model.

We demonstrate that pgACC myo-inositol is negatively associated with perceived paVA experience. It is well-known that stress-induced neural alterations likely depends on specific characteristics of the brain regions such as hippocampus (Heim et al., 2008). In a MRS study with major depressive disorder patients, blood cortisol showed significant negative correlation with myo-inositol levels and dehydroepiandrosterone-sulfate (DHEAS) displayed a significant negative relation with glutamate levels in right hippocampus, but not mPFC (Shirayama et al., 2017). Unlike the cortisol response, which decreased after repeated exposure to the stressor, the DHEAS response concentrations increase in response to both acute and chronic (repeated) stress in a primate study (Maninger et al., 2010). Thus, it is controversial the direct linkage between stress-induced excitotoxicity and the reduced myo-inositol in pgACC of subjects having PaVA exposure experience. Rather, this finding supports previous studies implicating myo-inositol in maltreatment or other biological or mechanistic alteration. In a mouse study, myo-inositol was decreased in the prefrontal cortex of mice that experienced social isolation (Corcoba et al., 2017). Few studies have investigated the effect of maltreatment *per se* on the chemical properties of the human brain, but several studies have investigated the relationship between the chemical properties of the brain and psychopathology. The myo-inositol to Cr ratio was reduced in adult depression patients with and without childhood trauma history (Coupland et al., 2005), while the NAA to Cr ratio was lower in maltreated subjects with posttraumatic stress disorder (PTSD; De Bellis et al., 2000). Decreased myo-inositol can be found in the prefrontal and cingulate regions of subjects with depressive disorder (Frey et al., 1998; Gruber et al., 2003; Coupland et al., 2005; Zheng et al., 2010), whereas other studies reported unchanged myo-inositol concentrations in depression patients (Auer et al., 2000; Farchione et al., 2002). Depression-related myo-inositol decrease was found in young adult subjects; thus, inconsistent results may be attributed to the confounding effect of age (Coupland et al., 2005; Zheng et al., 2010). Because myo-inositol measured by ^1^H-MRS is considered a biomarker for glia in many studies (Chang et al., 2013; Harris et al., 2015; Plitman et al., 2015), the decrease suggests altered glial function. Myo-inositol concentration is associated with maintenance of osmotic concentration (Rango et al., 2008) or cerebral metabolism (Weissenborn et al., 2007). Further, the hypo-osmolarity or its induced brain edema is a possible mechanism of the reduced myo-inositol in minimal hepatic encephalopathy which has no overt cognitive impairment (Kooka et al., 2016). Thus, biological changes in osmotic homeostasis, or brain metabolism may be also a possible explanation of our finding, the negative association of myo-inositol concentration in the pgACC with paVA.

We observed that several structural connectivities of the pgACC were negatively associated with perceived paVA experience. These connectivities include the connectivity of the pgACC with the right caudate and with the transverse temporal gyrus. Structural connectivity between the pgACC and the caudate can be considered part of the reward system. A task fMRI study on adults with childhood maltreatment history reported a blunted response to reward cues, decreased activity of the pallidum, and an association between childhood adversity and symptoms of anhedonia (Dillon et al., 2009). Other task fMRI studies have consistently reported decreased striatal responses to anticipated reward in maltreated individuals. Adolescent who experienced deprivation and adoption also showed reduced recruitment of the striatal reward system in an fMRI study (Mehta et al., 2010). A recent longitudinal fMRI study revealed that changes in ventral striatum activity were negatively correlated with the severity of emotional neglect (Hanson et al., 2015). These results are consistent with our findings. Blunted reward sensitivity makes people choose avoidance in approach-avoidance conflict to remain in a safe environment (Guyer et al., 2006; Teicher et al., 2016). The reduced connectivity in this study may be considered a potential protective adaptation to threatening environments, but further studies are needed to confirm this hypothesis.

Another novel observation that we observed was that weaker pgACC connectivity with the auditory cortex was related to paVA experience. Studies have reported changes in the cortical sensory region and tract associated with exposure to various types of maltreatment. The WM integrity of the left arcuate fasciculus associated with language processing was decreased in subjects with paVA (Choi et al., 2009). A similar voxel-based morphometry study revealed that young adults with repeated paVA have altered GM density in the primary auditory cortex within the superior temporal gyrus (Tomoda et al., 2011). Another study reported that decreased cortical thickness of the somatosensory cortex was associated with tactile sensation of the genital area in women with a childhood sexual abuse history (Heim et al., 2013). Another study of young adults who had suffered from witnessing interparental violence during their childhood revealed that these subjects had decreased WM integrity in the inferior longitudinal fasciculus, which connects visual association regions to temporal regions (Choi et al., 2012). Alterations of brain regions associated with primary sensory processing may reduce the specific type of distressing stimulus (i.e., maltreatment) and may be a potentially protective effect. However, further studies are needed to confirm this hypothesis.

We found that some patterns of connectivity of the pgACC with other regions are positively associated with VAQ score. From the perspective of the adaptation hypothesis (Belsky and Pluess, 2009; Teicher et al., 2016), this finding regarding pgACC connectivity has significant implications. This positive association of maltreatment-related brain changes with paVA suggests the experience-related plastic adaptation rather than diathesis-stress mechanism. Furthermore, as maltreatment-related experiences can be accepted as threats to survival, the strengthening of structure or functions of the brain may provide advantages in survival in aspect of the experience-related plastic adaptation. We found that structural connectivity between the pgACC and limbic regions was positively related to perceived paVA experience. The target regions of the connectivity included the amygdala and the entorhinal cortex. A recent study reported an inverse relationship between activities in the prefrontal region and the amygdala during the processing of a negative emotional stimulus (Amting et al., 2010). Increased functional connectivity between the rostral ACC and the amygdala is associated with emotional resilience (Kaiser et al., 2018). An association between WM connectivity and functional connectivity of these regions was reported (Lapate et al., 2016). Strong structural connectivity between the PFC region and the amygdala facilitates the easy processing of emotional stimuli and may protect against deleterious outcomes such as anxiety (Greening and Mitchell, 2015; Lapate et al., 2016). Therefore, our results suggest that stronger connection between the pgACC and the amygdala may be a consequence of developmental adaptation to maltreatment.

We also observed that paVA experience is associated with stronger connectivity between the pgACC and the inferior frontal region, such as the pars triangularis. This result is consistent with the framework of the plastic adaptation hypothesis. High cognitive performance, especially attention and alertness, is helpful for survival and adaptation. Several fMRI studies revealed a role of the cingulo-opercular network in the maintenance of attention-related cognitive processing (Dosenbach et al., 2008; Sadaghiani and D’Esposito, 2015; Coste and Kleinschmidt, 2016). Alertness can be defined as requiring cognitive resources, maintaining attention, and preparing to respond with less prior information. Those who experienced paVA have attempted to maintain their attention and prepare appropriate responses under repetitive negative stimuli. In this alert state, cingulo-opercular networks may be frequently induced. One possible interpretation is that in individuals who have experienced verbal abuse experience, an induced functional network, which is associated with negative emotional processing and maintenance of alertness, may enhance the structural network in the cingulate-limbic region and the cingulo-opercular region through plastic alterations, such as through a Hebbian process.

The connectivity of the pgACC with the right inferior temporal cortex and the left cuneus was positively associated with paVA experience. Although the significance of the association remains elusive, recent research can provide some clues. White matter integrity (assessed by fractional anisotropy) of the inferior fronto-longitudinal fasciculus (IFOF) in patients with trauma is correlated with emotional prosody and facial affect recognition (Schmidt et al., 2013; Genova et al., 2015), and the microstructure of the IFOF predicted emotional recognition performance (Unger et al., 2016). The pgACC connectivity may be part of the IFOF, which begins in occipital region and terminates in the frontal region via the temporal lobe. The IFOF plays roles in facial recognition, semantic memory, and emotional recognition. One possibility is that stronger IFOF connectivity is associated with more severe paVA experience to detect the caregiver’s emotion. Furthermore, considering the function of the cuneus and the inferior temporal cortex in visual recognition (Kravitz et al., 2013), strengthening this connectivity may be helpful for detecting emotional expressions, such as facial expressions.

The retrospective evaluation of paVA in a cross-sectional analysis of a cohort of subjects is a key limitation that risks the inclusion of recall bias in the results, although this bias could be related to the effect of emotional maltreatment on one’s inner model of perception of the world during his or her development. In addition, as we did not perform behavioral experiments associated with brain alterations, such as threat detection or reward processing, our neuroimaging results should not be interpreted as direct evidence of behavioral alterations. Subsequent work involving behavioral experiments may further elucidate the maltreatment-related adaptation hypothesis. Brain structures other than the pgACC, such as the corpus callosum and the hippocampus, are also associated with maltreatment (Teicher et al., 2010; Lee et al., 2018). Because we focused on pgACC-related regions as significant features for perceived paVA, the other structures were not considered. Although we cannot use interhemispheric connections as features of the model because of the limitations of our methodology, focusing on the medial frontolimbic circuit may be meaningful when considering the neurobiological background of maltreatment. However, it is necessary to investigate other prediction models using other seed regions, such as the hippocampus or the amygdala, in future studies. In addition, both low intensity of paVA and the modest sample size leads low variability and can reduce statistical power. Thus, further validation studies with independent larger samples including subjects with high intensity of paVA are also needed.

## Conclusion

Despite several limitations, this combined MRS and diffusion MRI study demonstrating the association of medial frontolimbic networks with a low level of paVA in healthy young adults indicates that exposure to emotional maltreatment, even at mild intensity, by key caregivers during childhood and adolescence can impact the development of brain circuits associated with reward, auditory, and emotional information processing.

## Author Contributions

BJ, DK and JY designed research; YP, DK and DS acquired diffusion image and MRS data; YP and DK preprocessed MRS data; DK and MK preprocessed diffusion image; DK and JY interviewed the participants for past history; DK performed statistical analysis; BJ and DK wrote the manuscript.

## Conflict of Interest Statement

The authors declare that the research was conducted in the absence of any commercial or financial relationships that could be construed as a potential conflict of interest.

## Footnotes

^1^https://fsl.fmrib.ox.ac.uk/fsl

^2^https://www.cmrr.umn.edu/downloads/mrspa/

^3^http://stnava.github.io/ANTs/

^4^https://surfer.nmr.mgh.harvard.edu/

^5^https://www.r-project.org/
