# Corrigendum: Anatomical and Neurochemical Correlates of Parental Verbal Abuse: A Combined MRS—Diffusion MRI Study

**DOI:** 10.3389/fnhum.2019.00456

**Published:** 2020-01-23

**Authors:** Dohyun Kim, Jae Hyun Yoo, Young Woo Park, Minchul Kim, Dong Woo Shin, Bumseok Jeong

**Affiliations:** ^1^Graduate School of Medical Science and Engineering, Korea Advanced Institute for Science and Technology (KAIST), Daejeon, South Korea; ^2^School of Electrical Engineering, Korea Advanced Institute of Science and Technology (KAIST), Daejeon, South Korea

**Keywords:** verbal abuse, frontolimbic circuit, magnetic resonance spectroscopy, diffusion tensor imaging, probabilistic tractography, pregenual anterior cingulate cortex, maltreatment

In the original article, we neglected to include the funder “Institute of Information & Communications Technology Planning & Evaluation (IITP) grant funded by the Korea government (MSIT)”, “No. 2017-0-00780” to “Bumseok Jeong.”

The authors apologize for this error and state that this does not change the scientific conclusions of the article in any way. The original article has been updated.

