# Supplementary material for: Anatomical and Neurochemical Correlates of Parental Verbal Abuse: A Combined MRS—Diffusion MRI Study
Source: Front Hum Neurosci. 2019 Jan 29;13:12. doi: 10.3389/fnhum.2019.00012 (PMC6361791; doi:10.3389/fnhum.2019.00012)
Supplement: Supplementary file 1 [file Data_Sheet_1.docx]

Supplementary Material

**Anatomical and neurochemical correlates of parental verbal abuse: A combined MRS – Diffusion MRI study**

Dohyun Kim^1^, Jae Hyun Yoo^1^, Y[oung Woo Park](https://onlinelibrary.wiley.com/action/doSearch?ContribAuthorStored=Park%2C+Young+Woo)^2^, Minchul Kim^1^, Dong Woo Shin^1^, and Bumseok Jeong*^1^

Corresponding author: Bumseok Jeong, M.D., Ph.D. Associate Professor, Director, Laboratory of Computational Affective Neuroscience and Development, Graduate School of Medical Science and Engineering, Korea Advanced Institute of Science and Technology, Daehak-ro 291, Daejeon, Korea Tel. +82-42-350-4245 (Office), E-mail: [bs.jeong@kaist.ac.kr](mailto:bs.jeong@kaist.ac.kr)

**Supplementary method**

**Partial least square regression (PLSR)**

In the standard linear regression for least square method for

Y=XB + $\varepsilon$

is represented as

B=${{(X}^{T}X)}^{-1}X^{T}Y$

where X is n x p matrix of independent variables and Y is n $\times$ 1 column vector of dependent variable (n: number of samples, p: number of independent variables).

In n < p case, $X^{T}X$is singular, therefore the least square method is not feasible

PLSR can overcome the problem by decomposing X and Y.

$X=TP^{T}+ \varepsilon_{1}$

$$Y=UQ^{T}+ \varepsilon_{2}$$

where T and Q are score (i.e component) vector, while P and Q are the loading matrix for X and Y respectively. In this study, X corresponds to 77 variables of connectivity and neurochemical features and Y corresponds to perceived parental verbal abuse. X and Y are decomposed as scores and loadings to explain maximum variance between X and Y. When the first score is found, the score is subtracted from both X and Y (Abdi, 2010). This process repeatedly depends on the number of components. We used PLS algorithms described in (Mevik and Wehrens, 2007) using pls package of R Software. We performed PLSR using two components, which are obtained from 77 variables sequentially (i.e. The first component for each subject was obtained from the matrix, and the second component for each subject was also obtained after subtracting the first component.).

**Jackknife approximation for the coefficients**

The significance of the features was assessed using jackknife approximation. The variances of features were estimated during cross validation. We performed the jackknife bootstrapping using the functions implemented in the *pls* R package. The detail result of the jackknife test is described in table S3 and figure S4. The features with 95% confidence intervals which either entirely above or below zero, were determined as significant features.

**Validation of the relationship between predicted VAQ and self-reported VAQ**

The relationship between predicted VAQ score and self-reported VAQ score was significant (r=0.53, p=1.2 X 10^-4^). However, two data points with high leverage were depicted in figure 4. The Cook’s distance of the two data point was outlier (> mean + 2SD). In order to diminish the effect of the outlier, we performed Spearman’s rank correlation analysis. The significance of this relationship was still retained (rho = 0.31, p-value=0.03).

**Supplementary tables and figures**

Table S1. Cramèr-Rao lower bounds (CRLB) range of each metabolite in all subjects (%)

| Metabolites | CRLB | Metabolites | CRLB | Metabolites | CRLB | Metabolites | CRLB |
| --- | --- | --- | --- | --- | --- | --- | --- |
| Alanine | 43 ‑ 999 | Glucose | 13 ‑75 | Myo-inositol | 3 ‑ 5 | Phosphor-ethanolamine | 12 ‑ 53 |
| Aspartate | 12 ‑ 127 | Glutamine | 10 ‑ 46 | Scyllo-inositol | 14 ‑ 999 | N-acetyl-aspartate | 2 ‑ 4 |
| Ascorbate | 17 ‑ 999 | Glutamate | 3 - 11 | Lactate | 18 ‑ 255 | N-acetylaspartate glutamate | 92 - 999 |
| Creatine | 6 ‑ 40 | Glycerylphos-phorycholine | 6 ‑ 26 | Phosphor-creatine | 7 ‑ 63 | Taurine | 15 ‑ 999 |
| GABA | 16 ‑ 999 | Glutathione | 9 ‑ 61 | Phosphor-choline | 26 ‑ 173 | Macromolecule^*^ | 3 ‑ 7 |
| CRLB: Cramèr-Rao lower bounds  * The macromolecule baseline was also fitted to ensure accurate measurement of the other metabolites. | | | | | | | |

Table S2. List of brain regions of interest (ROIs) used as target regions for the probabilistic tractography. The last four ROIs were excluded from the analysis because the ROIs overlapped the pgACC volume of interest, which was used as the seed region (shown in grey).

| **L, R accumbens area** | **L, R amygdala** |
| --- | --- |
| **L, R caudal anterior cingulate** | **L, R caudal middle frontal gyrus** |
| **L, R caudate** | **L, R cuneus** |
| **L, R entorhinal** | **L, R fusiform** |
| **L, R hippocampus** | **L, R inferior parietal lobule** |
| **L, R inferior temporal gyrus** | **L, R insula** |
| **L, R isthmus cingulate cortex** | **L, R lateral occipital gyrus** |
| **L, R lateral orbitofrontal** | **L, R lingual** |
| **L, R middle temporal gyrus** | **L, R pallidum** |
| **L, R paracentral** | **L, R parahippocampal** |
| **L, R pars opercularis** | **L, R pars orbitalis** |
| **L, R pars triangularis** | **L, R pericalcarine** |
| **L, R postcentral** | **L, R posterior cingulate cortex** |
| **L, R precentral** | **L, R precuneus** |
| **L, R putamen** | **L, R rostral middle frontal gyrus** |
| **L, R superior frontal gyrus** | **L, R superior parietal lobule** |
| **L, R superior temporal gyrus** | **L, R supramarginal gyrus** |
| **L, R thalamus** | **L, R transverse temporal** |
| L, R medial orbitofrontal | L, R rostral anterior cingulate cortex |

Table S3 Features that make reliable contribution to the partial least square regression model for parental verbal abuse

|  | Coefficient 95% confidence intervals | | |
| --- | --- | --- | --- |
| Right Caudate | -0.78094 | -1.53667 | -0.02521 |
| Right amygdala | 0.807987 | 0.150778 | 1.465196 |
| Left cuneus | 0.764134 | 0.025173 | 1.503095 |
| Left pars triangularis | 0.702842 | 0.2587 | 1.146985 |
| Right entorhinal | 0.620079 | 0.135289 | 1.104869 |
| Right inferior temporal | 0.780662 | 0.112115 | 1.449208 |
| Right transverse temporal | -0.71998 | -1.40417 | -0.0358 |
| Myo-inositol | -0.76774 | -1.43318 | -0.1023 |

Figure S1. Histogram of the root mean square (RMS) of motion (left) and the LCModel signal-to-noise ratio (SNR) (right) of 49 subjects. Three participants corresponding to outliers were excluded from the analysis. Two subjects were excluded because of excessive motion (RMS of motion >3), and one subject was excluded due to poor LCModel fitting (SNR <10).


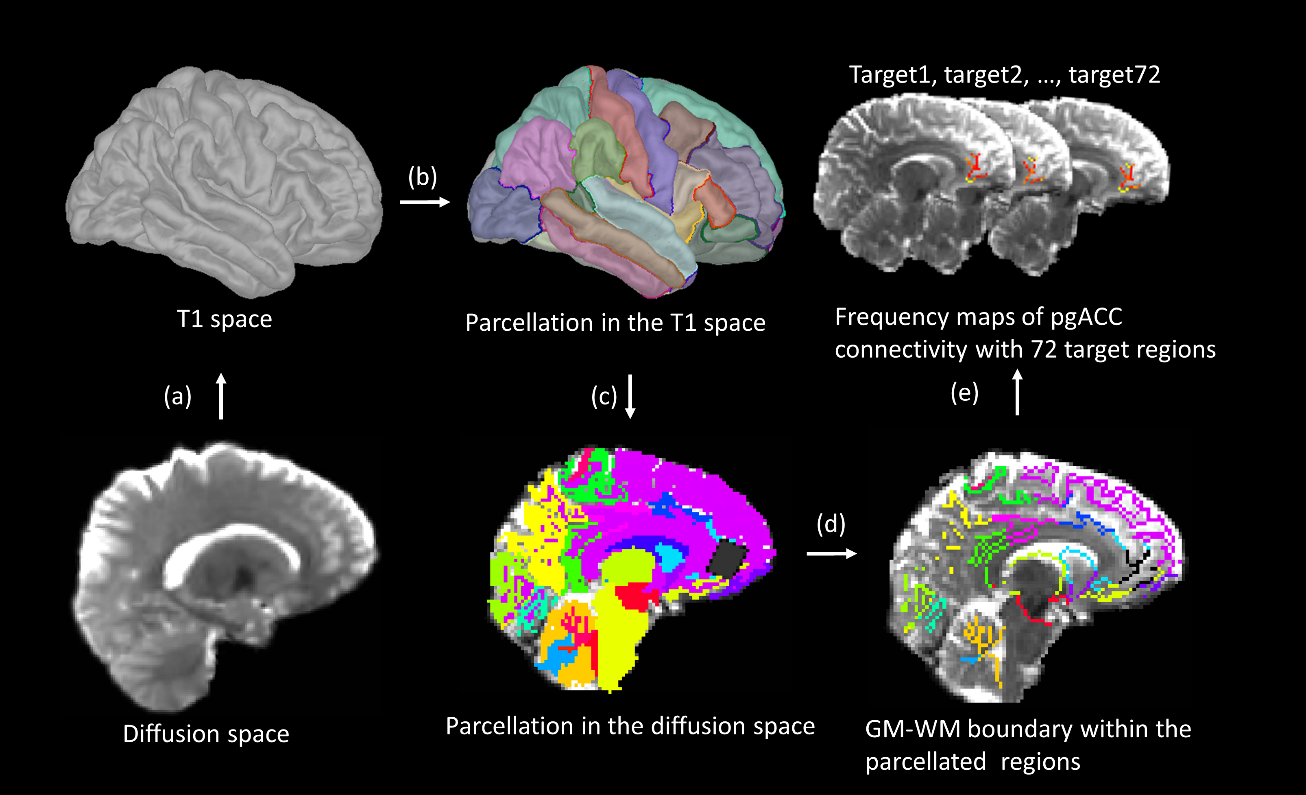


Figure S2. Processing steps for pgACC connectivity. (a) Diffusion images are transformed to the T1 space using Advanced Normalization Tools (b) Structural images are processed via the Freesurfer pipeline to obtain cortical and subcortical segmentation. (c) The cortical and subcortical parcellated images are reverse-transformed to the diffusion space. The black rectangle represents the volume of interest (VOI) of magnetic resonance spectroscopy (MRS). (d) The boundaries between the grey matter and the white matter within the parcellated regions are obtained. (e) The pgACC connectivity is defined as average value within the connectivity likelihood map of seed – target connectivity derived from probabilistic tractography (pgACC: pregenual anterior cingulate cortex, GM: grey matter, WM: white matter)


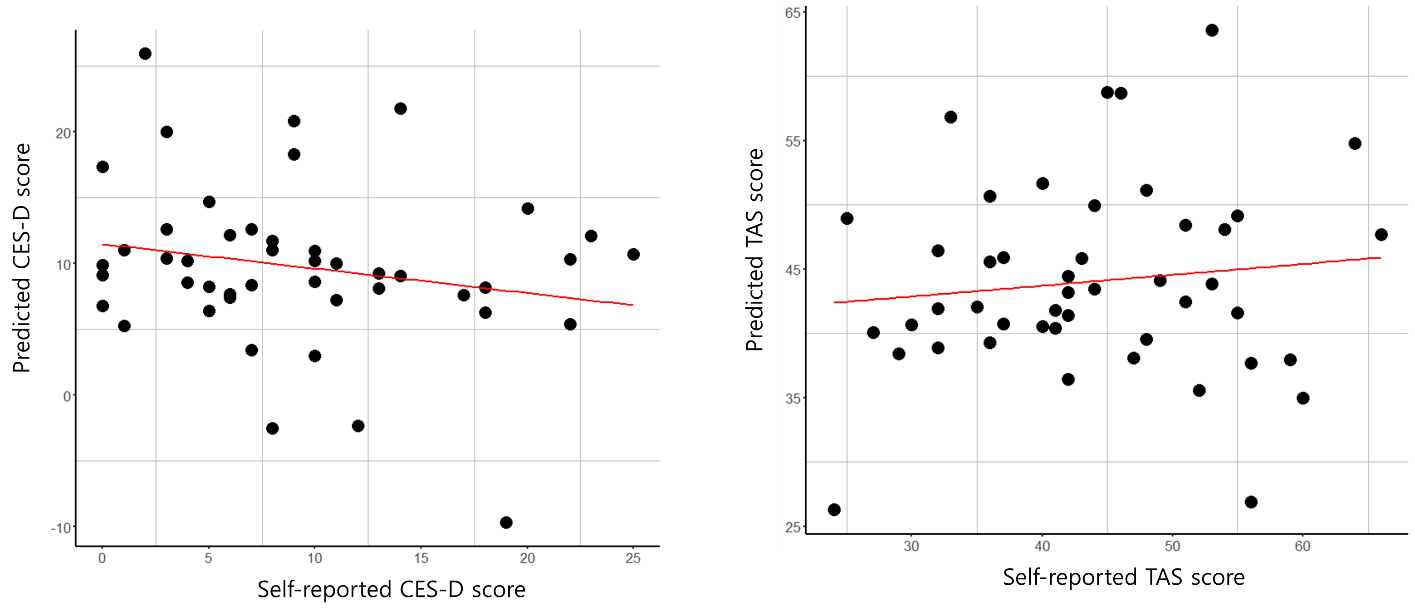


Figure S3. Scatter plot of self-reported scores and predicted scores of the Center for Epidemiological Studies – Depression scale (CES-D, left) and the Toronto alexithymia scale (TAS, right) from the partial least square regression model. Predicted values were obtained using leave-one-out testing. We perform a correlation analysis between the predicted values and the self-reported values. The correlation coefficients were -0.20 (p-value = 0.17) and 0.12 (p-value = 0.44) for CES-D and TAS, respectively.

Figure S4. PLSR coefficients from the jackknife approximation. The bars in line indicate two standard errors of estimation. Red represents significantly positive coefficients, and blue represents significantly negative coefficients.


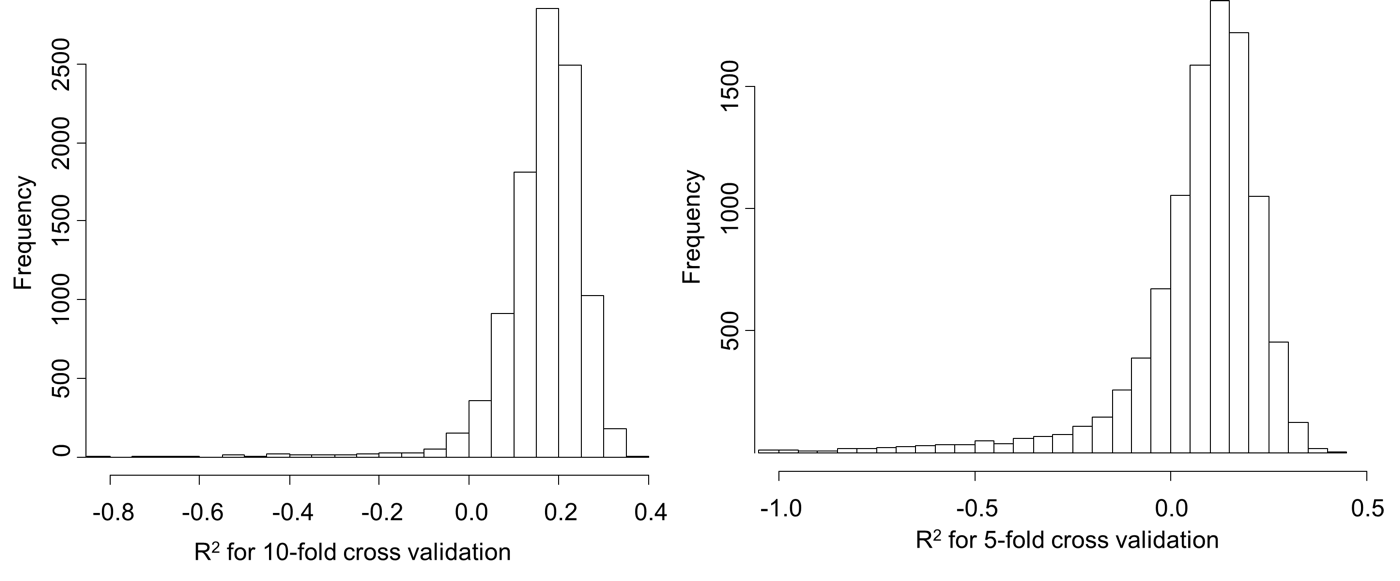


Figure S5. Histograms of the cross-validated prediction R^2^ for different cross-validation tests. We perform 10,000 cross-validation iterations for the 10-fold (left) and five-fold scenarios. For each iteration, the cross-validated prediction R^2^ is obtained. For 10-fold cross-validation, 9636 R^2^ values were above 0, and for 5-fold cross-validation, 7818 values were above 0. R^2^ values less than -1 (3/10000 for 10-fold and 115/10000 for five-fold) were excluded from the histogram for visualization purposes.

**References**

Abdi, H. (2010). Partial least squares regression and projection on latent structure regression (PLS Regression). *Wiley Interdisciplinary Reviews: Computational Statistics* 2(1)**,** 97-106. doi: 10.1002/wics.51.

Mevik, B.H., and Wehrens, R. (2007). The pls package: Principal component and partial least squares regression in R. *Journal of Statistical Software* 18(2)**,** 1-23. doi: 10.18637/jss.v018.i02.
